# Supplementary material for: The Construction and Exploration of a Comprehensive MicroRNA Centered Regulatory Network in Foxtail Millet (Setaria italica L.)
Source: Front Plant Sci. 2022 May 6;13:848474. doi: 10.3389/fpls.2022.848474 (PMC9121102; doi:10.3389/fpls.2022.848474)
Supplement: Supplementary file 5 [file Data_Sheet_5.zip › Sit-miR319_Seita.4G165500.1_671_TPlot.pdf]

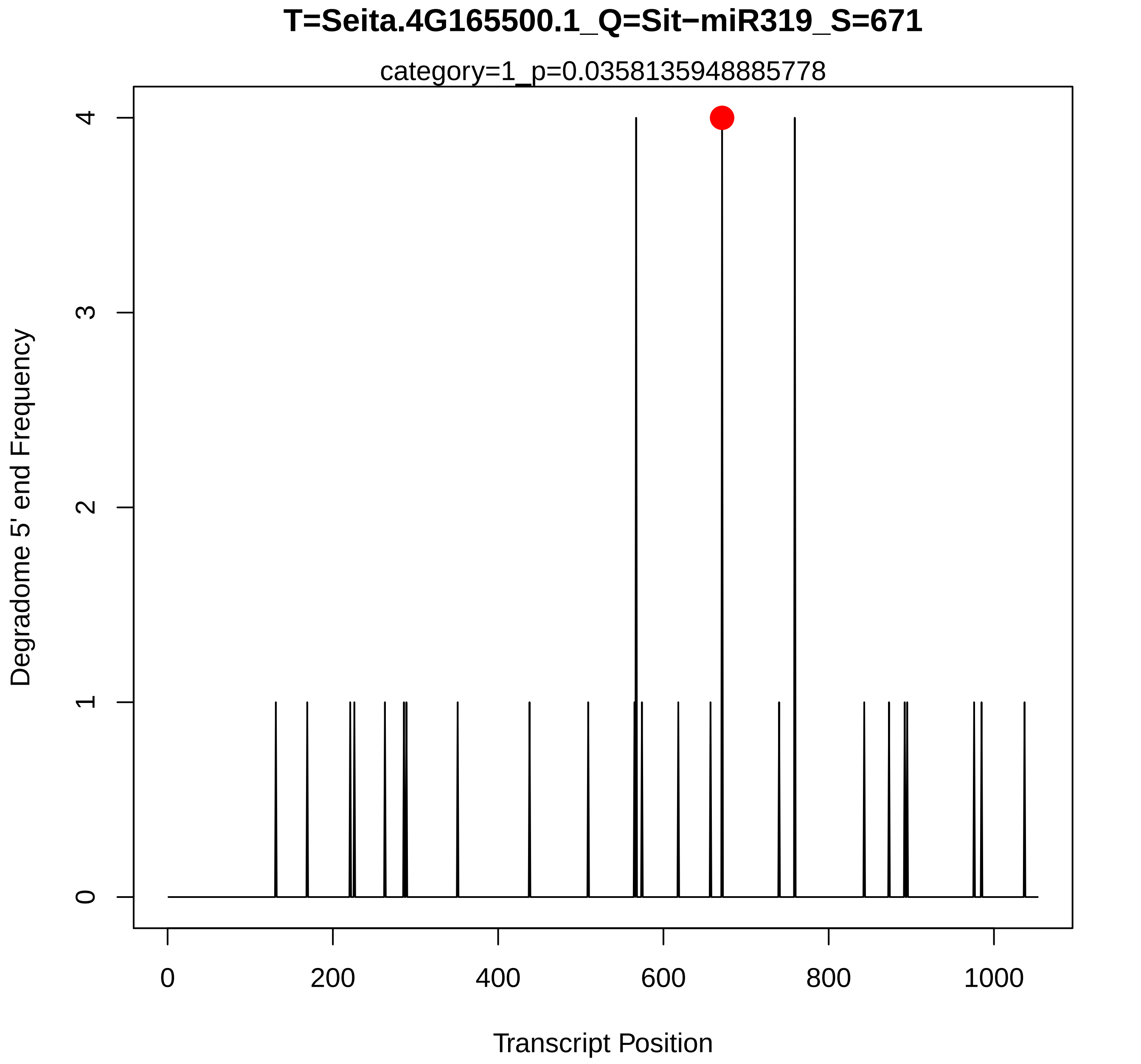
T=Seita.4G165500.1_Q=Sit−miR319_S=671
category=1_p=0.0358135948885778
Degradome 5' end Frequency
0
1
2
3
4
0
200
400
600
800
1000
Transcript Position
